# Opioid use and adverse health effects in breast cancer survivors

**DOI:** 10.1093/oncolo/oyae270

**Published:** 2024-10-14

**Authors:** Reina Haque, Lie Hong Chen, Jiaxiao Shi, Zheng Gu, Moira Brady-Rogers, Rowan T Chlebowski, Rulin C Hechter

**Affiliations:** Department of Research & Evaluation, Kaiser Permanente Southern California, Pasadena, CA 91101, United States; Department of Health Systems Science, Kaiser Permanente Bernard J. Tyson School of Medicine, Pasadena, CA 91101, United States; Department of Research & Evaluation, Kaiser Permanente Southern California, Pasadena, CA 91101, United States; Department of Research & Evaluation, Kaiser Permanente Southern California, Pasadena, CA 91101, United States; Department of Research & Evaluation, Kaiser Permanente Southern California, Pasadena, CA 91101, United States; Alive & Well Women, South Pasadena, CA 91030, United States; The Lundquist Research Institute for Biomedical Innovation at UCLA-Harbor & Division of Medical Oncology and Hematology, Torrance, CA 90502, United States; Department of Research & Evaluation, Kaiser Permanente Southern California, Pasadena, CA 91101, United States; Department of Health Systems Science, Kaiser Permanente Bernard J. Tyson School of Medicine, Pasadena, CA 91101, United States

**Keywords:** opioids, breast cancer; survivorship, adverse effects, falls, fractures, cardiovascular events, chronic obstructive pulmonary disease

## Abstract

**Background:**

Little information exists on adverse effects related to opioid use in breast cancer survivors following active cancer treatment, and no studies included an age-matched comparison group. Thus, we examined opioid use and risk of falls, fractures, lung problems, and cardiovascular events in breast cancer survivors in the years following active cancer treatment along with a comparison group.

**Methods:**

We conducted a longitudinal cohort study 33 989 breast cancer survivors and 157 609 age-matched women without cancer. Rates of adverse events, and multivariable hazards ratios for association between opioid use and the adverse health effects were calculated.

**Results:**

Women with breast cancer had greater opioid use (60% vs 48%); longer median opioid duration (18 vs 16 days); and were prescribed stronger opioids than the matched cohort over 5.6 median years of follow-up. In multivariable models, the risk of falls was 12% higher (HR, 95% CI, 1.12 [1.07-1.17]), and fracture risk was 56% (HR = 1.56 [1.48-1.65]) greater in women with breast cancer who used opioids vs the matched cohort unexposed to opioids. In an analysis restricted to women with breast cancer, opioid use was strongly associated with the risk of falls (HR = 1.74 [1.63-1.85]); fractures (HR = 2.10 [1.95-2.27]); lung problems (HR = 1.53 [1.43-1.64]); and cardiovascular events (HR = 1.70 [1.39-2.08]) than opioid non-use.

**Conclusions:**

After active cancer treatment, opioid use and high dosage use were common in breast cancer survivors, and were associated with increased risk for falls, lung problems, fractures, and cardiovascular events. Findings underscore the need for careful monitoring of opioid use in these survivors and the exploration of alternative pain management strategies.

## Teaser

This study examined opioid use and the risk of falls, fractures, lung problems, and cardiovascular events in breast cancer survivors in the years following active cancer treatment, along with a comparison group.

**Implications for practice**

This study found that opioid use in the years beyond active cancer treatment was common in women with breast cancer, and associated with an increased risk of adverse health effects and injuries than in matched women without cancer (comparison group) unexposed to opioids. Further, steps for opioid risk reduction at the pharmacy or healthcare provider level may help to mitigate such risks.

## Background

Worldwide, women with breast cancer accounted for nearly one-quarter of all cancer cases diagnosed in 2020, and the disease ranked first for cancer-related deaths.^1^ In the United States, there are over 3 million breast cancer survivors, and almost 90% of them receive prescription opioids as the first-line pain therapy after surgery and continue to use them long after, and a substantial fraction become chronic users.^2-4^ Breast cancer survivors may experience polydrug toxicity associated with chronic use of opioids and psychotropic drugs and, hence may be more susceptible to developing opioid dependence and other adverse health outcomes. However, limited data exist on consequent health outcomes in breast cancer survivors related to opioid use and misuse, and the studies that examined opioid-related adverse health effects often excluded patients with cancer.^5-13^

Hence, a death of information exists on adverse effects related to opioid use in patients with breast cancer following their active cancer treatment, and no studies included an age-matched comparison group.^14^ Some of the existing knowledge stems from studies based on advanced-cancer related pain or during palliative care.^15,16^ Women diagnosed with early-stage breast cancer have a 10-year survival rate of 83%,^17^ and given the growing number of breast cancer survivors, it is of great public health significance to determine the adverse health outcomes associated with opioid use in this vulnerable population. In parallel, in response to the growing opioid crisis in the United States, access to appropriate pain management drugs in cancer survivors is becoming increasingly difficult.^16^ For many cancer survivors, national oncology guidelines support use of opioids for effective pain management.^16^ Given the large number of breast cancer survivors suffering from chronic pain, examining the safety or potential adverse effects of opioid use is urgent.

To address these research gaps, we conducted a longitudinal cohort study of a diverse group of patients with breast cancer in a large non-profit integrated healthcare system in southern California. The aim of this study was to estimate population-based rates of serious health consequences associated with prescription opioid use in breast cancer survivors in the years after active cancer treatment, and compare the risk of these events in a group of age-matched women without cancer to assess if cancer survivorship care plans should incorporate long-term monitoring of prescription pain medications. Specifically, we examined the risk of serious health consequences of opioid use in the years after active cancer treatment. We specifically examined falls, fractures, lung problems (pneumonia, chronic obstructive pulmonary disease), and cardiovascular events.^5-14^ We hypothesized that breast cancer survivors would be more likely to have used opioids and have a greater risk of the adverse events as compared with an age-matched cohort.

## Methods

### Design, data sources, and setting

This longitudinal cohort study was conducted at Kaiser Permanente Southern California (KPSC), a vertically integrated healthcare system with 17 hospitals, over 200 clinics, a network of nearly 8000 physicians practicing primary care and specialties, and approximately 4.7 million patient members. The longitudinal retrospective cohort study is the strongest observational study design in epidemiology because prospective studies (and randomized trials) are often not feasible or cost prohibitive, vulnerable to attrition, and usually have small samples and shorter follow-up.^18^ At KPSC, patients receive virtually all their healthcare within the system. Electronic health records captured information on membership, tumor registry, pharmacy, laboratory, outpatient, inpatient, and emergency department diagnosis codes, linked census information, and death data. Deaths were identified from a database that incorporates information from the health plan membership, and state and national death databases. The health plan’s National Cancer Institute’s Surveillance, Epidemiology, and End Results (SEER) affiliated cancer registry was used to identify women with breast cancer, their tumor characteristics and adjuvant treatments. The KPSC institutional review board (IRB) reviewed and approved this study; written and verbal informed consent was waived by the IRB. We followed the Strengthening the Reporting of Observational studies in Epidemiology (STROBE) guidelines.

### Study groups and follow-up

We assembled a cohort of adult women (≥18 years) diagnosed with in situ, localized, and regional breast cancer from 2009 to 2019, with at least 1 year of survivorship after their initial breast cancer diagnosis. The index date was the point of cancer survivorship, being 1 year after the breast cancer diagnosis. We also assembled an age-matched comparison group of women without cancer from the KPSC membership. Comparisons were selected using the same enrollment criteria and individually matched based on sex, exact age, and medical center without replacement in an approximately 1:5 ratio. Comparisons were assigned an index date corresponding to their matched case. Subjects were excluded if they were not continuously enrolled for 12 months or died prior to the index date. The study cohort was followed from the index date until study outcomes, membership disenrollment, death, or December 31, 2021 (end of study), whichever occurred first.

### Outcomes

We examined incident cardiovascular events (cardiac arrhythmia, acute myocardial infarction, and stroke); lung problems (chronic obstructive pulmonary disease, pneumonia); fractures; and falls requiring medical attention from the index date through the end of follow-up. These events were identified using from in-patient or outpatient settings; inpatient codes were prioritized, and at least 2 records of outpatient codes were required (see Table S1 for ICD-9 and ICD10 diagnostic codes). The events were examined separately, and as a composite, based on any event that occurred the earliest.

### Opioid use

We extracted information on prescription opioid use from the health plan’s pharmacy dispensing databases (generic name, dates, days supplied, dosage). Opioids included: hydrocodone, hydromorphone, oxycodone, oxymorphone, tramadol, morphine, and fentanyl. We examined opioid use from the index date through the end of each patient’s follow-up. Thus, the time window for opioid use assessment started the day after the index date and ended the day before each patient’s censoring event (ie, until patient developed the study outcome, disenrolled from the health plan, died, or reached the study’s end [December 31, 2021], whichever occurred first).

To avoid overestimating opioid use for acute pain, at least 2 continuous opioid prescriptions were required. The duration of use was calculated as cumulative days supplied. Annualized duration was also calculated. We also estimated the maximum daily morphine milligram equivalents (MME) based on prescription strength, days supplied, and a MME conversion factor.^19^ We also determined persistent opioid use during follow-up, defined as continuously receiving ≥90 days’ supply of opioids (allowing for 15-day gaps) during patients’ entire follow-up period.^20^ History of use of these opioids (yes/no) in the year prior to index was included as a covariate.

### Covariates

We extracted a comprehensive set of covariates from the electronic health records in the year prior to the index date (ie, baseline): age; race/ethnicity; geocoded median annual household income and education at census tract level; body mass index (BMI kg/m^2^); smoking status; substance use disorder history; depression; and anxiety. If multiple records of the covariate existed, we used the one closest to the index date. We also extracted current use of covariate prescription medications during follow-up including psychiatric medications (antidepressants, anxiolytics, sedatives/sleep aids); and other prescribed pain medications (non-steroidal anti-inflammatory drugs [NSAIDs]; cannabinoids; gabapentin). Baseline Elixhauser Comorbidity Index (ECI) was calculated; we excluded cancer diagnosis in the modified ECI calculation for breast cancer survivors.^21^ We also calculated the annualized number of outpatient visits. For breast cancer survivors, we also extracted tumor characteristics and primary treatments (SEER summary stage; year of diagnosis; surgery type; adjuvant chemotherapy, radiation, and endocrine therapy) from the cancer registry. In general, we had low percent missing for most study variables (except for smoking status and BMI); hence, we included a category for missing values in the multivariable analyses to maintain the full sample size.

### Statistical analysis

Differences in baseline sociodemographics and clinical characteristics were compared between the 2 groups: breast cancer survivors and matched comparisons. The chi-square test was used to compare categorical variables. Continuous variables were evaluated via medians and interquartile ranges given non-normally distributed data, and compared with Wilcoxon-Mann-Whitney test.

Incidence rates (per 1000 person-years) and 95% CI were calculated for each study outcome by opioid use status for both study groups. Cumulative incidence rates were calculated using the cumulative incidence function by opioid use, accounting for competing risk of overall death and censoring individuals at the end of follow-up.

Two sets of multivariable Cox proportional hazards modeling were conducted to study the association between opioid use and adverse events incorporating time-dependent opioid use and concurrent medication use (ie, 0 up to the start date, 1 thereafter) during follow-up. The first model examined risk of adverse outcomes associated with opioid use in breast cancer survivors and matched comparisons adjusted for aforementioned covariates that were common to both groups. Proportional hazard assumption checks were implemented for all variables; we found no evidence of assumption violations. Variables were checked for collinearity. If variable had missing values, a missing category was used (eg, for smoking, geocoded income, and BMI). The second model was restricted to breast cancer survivors. The second model followed a similar analytic strategy, but included tumor characteristics and cancer treatment variables, in addition to covariates in the first model. All statistical tests were 2-sided and assessed at *P* < .05 level. We used SAS 9.4 for data extraction and analyses.

## Results

### Breast cancer survivors and comparison characteristics

We identified 37 061 women with early stage (in situ; local; regional) breast cancer from 2009 to 2019; of these 3051 were excluded because they were not continuously enrolled for 12 months or died prior to index date. Thus, the breast cancer survivor cohort included 33 989 women (median age: 62 years) and 157 609 comparison cohort (median age: 62 years). The combined cohort was diverse: 12% Asian/Pacific Islander; 12% Black; 29% Hispanic; 44% non-Hispanic White, and 3% other/mixed (Table 1). Breast cancer survivors were more likely to have obesity (38% vs 33%), be former smokers (23% vs 17%), and have more comorbidities (ECI 5+: 23% vs 10%) than comparison women, respectively (*P* < .001 for all variables).

**Table 1.** Demographic and clinical characteristics of breast cancer survivors and age-matched comparison cohort.

	Total (*N* = 191 598) *N* (%)	Breast cancer (*N* = 33 989) *N* (%)	Comparison (*N* = 157 609) *N* (%)	*P*-value^*^
**Age at index (years)**				.056
<40	6603 (3.4)	1142 (3.4)	5461 (3.5)	
40-64	102 084 (53.3)	17 946 (52.8)	84 138 (53.4)	
65+	82 911 (43.3)	14 901 (43.8)	68 010 (43.2)	
**Race/ethnicity**				<.001
Non-Hispanic White	84 293 (44.0)	17 030 (50.1)	67 263 (42.7)	
Black	22 657 (11.8)	4176 (12.3)	18 481 (11.7)	
Hispanic	56 256 (29.4)	7533 (22.2)	48 723 (30.9)	
Asian/Pacific Islander	23 405 (12.2)	4802 (14.1)	18 603 (11.8)	
Mixed/other	4987 (2.6)	448 (1.3)	4539 (2.9)	
**Geocoded median household income**				<.001
≤ $40 000	23 422 (12.2)	3979 (11.7)	19 443 (12.3)	
$40 001-$65 000	65 529 (34.2)	11 399 (33.5)	54 130 (34.3)	
$65 001+	102 493 (53.5)	18 588 (54.7)	83 905 (53.2)	
Unknown	154 (0.1)	23 (0.1)	131 (0.1)	
**Elixhauser comorbidity index (ECI)**				<.001
0	43 596 (22.8)	2918 (8.6)	40 678 (25.8)	
1	39 701 (20.7)	5583 (16.4)	34 118 (21.6)	
2	33 449 (17.5)	6840 (20.1)	26 609 (16.9)	
3	24 742 (12.9)	6155 (18.1)	18 587 (11.8)	
4	16 380 (8.5)	4640 (13.7)	11 740 (7.4)	
5+	24 296 (12.7)	7853 (23.1)	16 443 (10.4)	
Unknown	9434 (4.9)	0 (0.0)	9434 (6.0)	
**Body mass index (kg/m** ^**2**^ **)**				<.001
<18.5 (underweight)	2747 (1.4)	485 (1.4)	2262 (1.4)	
18.5-24.9 (healthy)	50 833 (26.5)	9751 (28.7)	41 082 (26.1)	
25-29.9 (overweight)	56 010 (29.2)	10 896 (32.1)	45 114 (28.6)	
≥30 (obese)	65 263 (34.1)	12 833 (37.8)	52 430 (33.3)	
Unknown	16 745 (8.7)	24 (0.1)	16 721 (10.6)	
**Smoking history**				<.001
Never	118 674 (61.9)	23 272 (68.5)	95 402 (60.5)	
Current	8897 (4.6)	1311 (3.9)	7586 (4.8)	
Former	33 822 (17.7)	7785 (22.9)	26 037 (16.5)	
Unknown	30 205 (15.8)	1621 (4.8)	28 584 (18.1)	
**History of adverse events before index date**				
Falls	7565 (3.9%)	1034 (3.0%)	6531 (4.1%)	<.001
Lung problems	9209 (4.8%)	1838 (5.4%)	7371 (4.7%)	<.001
Fractures	5813 (3.0%)	836 (2.5%)	4977 (3.2%)	<.001
Cardiovascular events	1193 (0.6%)	252 (0.7%)	941 (0.6%)	.002

^*^Based on chi-square test (2-sided).

### Opioid use in breast cancer survivors and comparisons during follow-up

**Figure 1.**
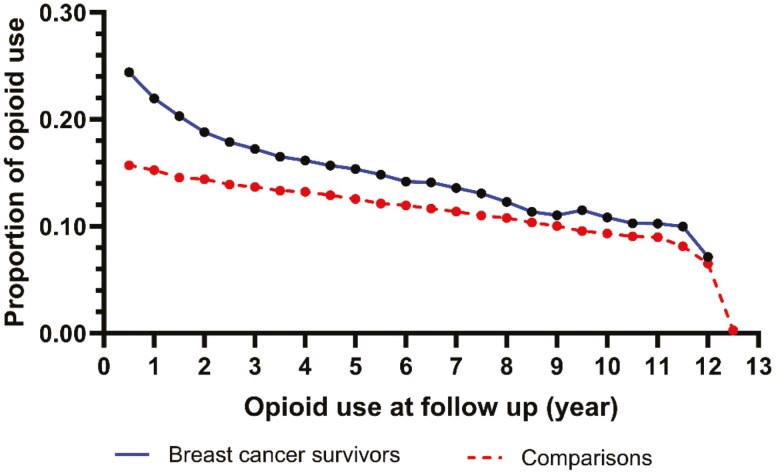
Percentage of opioid use during study follow-up by breast cancer survivors and matched comparison cohort.

Breast cancer survivors and the matched comparison cohort were followed for a maximum of 12 years (median: 5.6 years). Table 2 presents the distribution of prescription opioid use and other concurrent pain medications during follow-up in breast cancer survivors and comparisons. Breast cancer survivors had greater exposure to opioids than comparisons (Figure 1), respectively: overall percent using opioids (60% vs 48%); longer median opioid duration (18 vs 16 days); and higher median maximum daily MME (54.1 vs 44.3) (*P* < .001 for all variables). Further, breast cancer survivors were more likely to use opioids with greater strength than comparisons such as tramadol (19% vs 16%); hydrocodone (34% vs 35%); oxycodone (14% vs 10%); respectively (*P* < .001 for all variables). Breast cancer survivors were also more likely to use morphine and fentanyl than the comparison cohort, but such use was rare. Reasons for opioid use in the electronic records were not available, however, *n* = 712 (2.1%) of breast cancer survivors had chemotherapy induced neuropathy.

**Table 2.** Exposure to opioids, other pain medications, and psychiatric drugs among breast cancer survivors and age-matched comparison cohort.

	Total	Breast cancer	Comparison	*P*-value^*^
	(*N* = 191 598)	(*N* = 33 989)	(*N* = 157 609)	
Opioid ever use	95 981 (50.1%)	20 311 (59.8%)	75 670 (48.0%)	<.001
Opioid use (days)**				<.001
Median (IQR)	16.0 (6.0, 60.0)	18.0 (8.0, 60.0)	16.0 (6.0, 60.0)	
Annualized opioid use, days^**^				<.001
Median (IQR)	2.9 (1.2, 10.6)	3.4 (1.4, 11.8)	2.7 (1.1, 10.3)	
Opioid persistent use^**^	11142 (11.6%)	2285 (11.3%)	8857 (11.7%)	.073
Maximum daily MME**	31.3 (20.8, 60.0)	37.5 (25.0, 62,5)	30.0 (20.0, 50.0)	<.001
**Opioid types**				
Hydrocodone	69 351 (36.2%)	15 323 (45.08%)	54 028 (34.28%)	<.001
Tramadol	32 094 (16.8%)	6330 (18.6%)	25 764 (16.3%)	<.001
Other	28 417 (14.83%)	5361 (15.77%)	23 056 (14.63%)	<.001
Oxycodone	20 215 (10.55%)	4882 (14.36%)	15 333 (9.73%)	<.001
Morphine	6648 (3.47%)	1749 (5.15%)	4899 (3.11%)	<.001
Hydromorphone	3906 (2.04%)	1072 (3.15%)	2834 (1.8%)	<.001
Fentanyl	1497 (0.78%)	467 (1.37%)	1030 (0.65%)	<.001
Oxymorphone	64 (0.03%)	13 (0.04%)	51 (0.03%)	.590
Opioid use in year before index date	68 818 (35.9%)	31 652 (93.1%)	37 166 (23.6%)	<.001
**Other medications (prescribed)**				
Cannabinoids	573 (0.3%)	211 (0.6%)	362 (0.2%)	<.001
NSAIDS	83 520 (43.6%)	15 228 (44.8%)	68 292 (43.3%)	<.001
Gabapentin	29 452 (15.4%)	6425 (18.9%)	23 027 (14.6%)	<.001
Antidepressants	63 607 (33.2%)	12 716 (37.4%)	50 891 (32.3%)	<.001
Anxiolytics	12 663 (6.6%)	3634 (10.7%)	9029 (5.7%)	<.001
Sedatives/sleep aids	45 588 (23.8%)	9985 (29.4%)	35 603 (22.6%)	<.001

^*^ *P* values were 2-sided and based on chi-square test for categorical variables and Kruskall–Wallis test for medians.

^**^Based on patients who used opioids.

^***^Subclasses were grouped together to ensure robust statistical analysis for the study outcomes.

Persistent opioid use, defined as continuous use of ≥90 days, was similar among breast cancer survivors and the comparison cohort (about 11%). Prescription NSAIDS use was similar in both groups (about 44%). Gabapentin use was slightly greater in breast cancer survivors (19% vs 15%). The use of cannabinoids was low in both groups (0.6% in breast cancer survivors vs 0.2% in comparisons). Breast cancer survivors were more likely to use psychiatric drugs including anxiolytics (11% vs 6%); antidepressants (37% vs 32%); and sedatives or sleep aids (29% vs 23%) than the comparison group, respectively (*P* < .001 for all variables).

### Incidence rates of adverse events by opioid in breast cancer survivors and comparison

**Figure 2.**
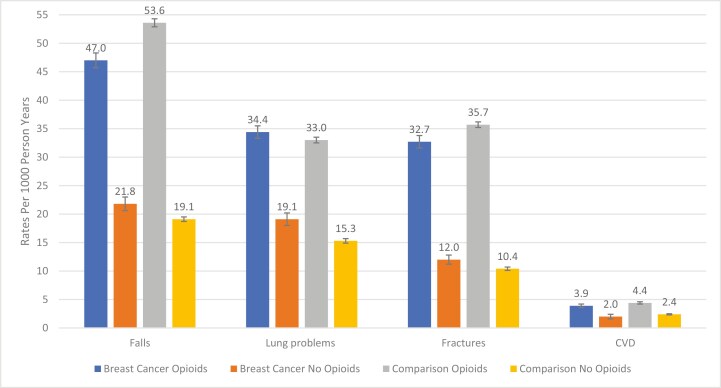
Person-year rates per 1000 of adverse events in breast cancer survivors and matched comparisons.

Overall, falls were the most common event, followed by lung problems, fractures, and cardiovascular events in our study population (Figure 2). In both breast cancer survivors and the comparison cohort, the cumulative incidence of adverse events during follow-up was dramatically higher in those who used opioids compared to those who did not use opioids. For example, in breast cancer survivors, the cumulative incidence rate of falls was twice as high in opioid users (47.0/1000 PY, 95% CI, 45.7-48.3) vs non-opioid users (21.8/1000 PY, 95% CI, 20.6-23.0). The cumulative incidence for the composite outcome was doubled in breast cancer survivors who used opioids (90.9/1000 PY, 95% CI, 89.0-92.9) than in non-users (45.9/1000 PY, 95% CI, 44.2-47.8). We found the same pattern in the comparison cohort, with cumulative incidence rates of the composite outcome being higher in the opioid use group (99.0/1000 PY, 95% CI, 98.0-100.0) than in non-users (39.4/1000 PY, 95% CI, 38.8-40.0).

### Multivariable analyses in breast cancer survivors and comparison group

**Figure 3.**
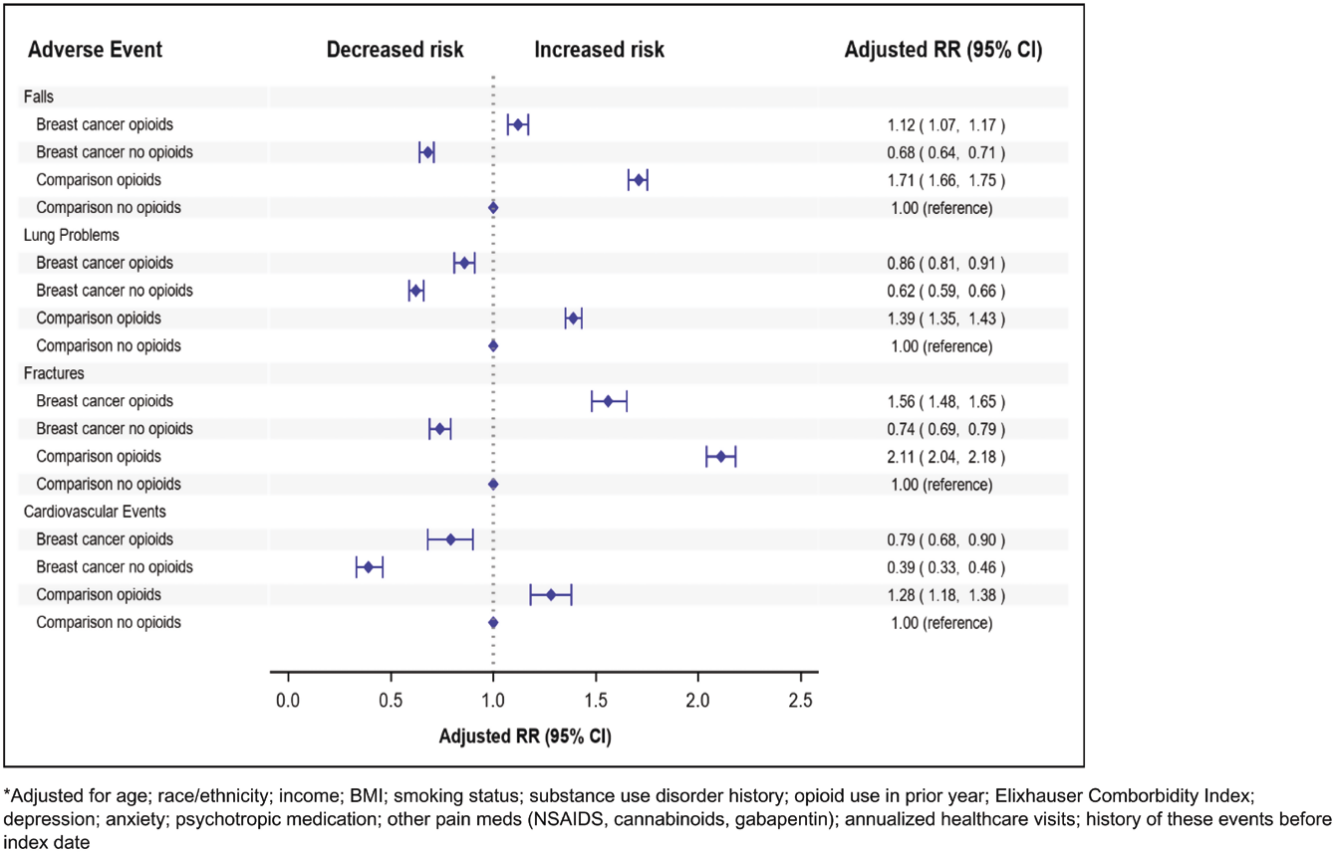
Adjusted association of adverse outcomes in breast cancer survivors & matched comparisons by opioid use during follow-up.

**Figure 4.**
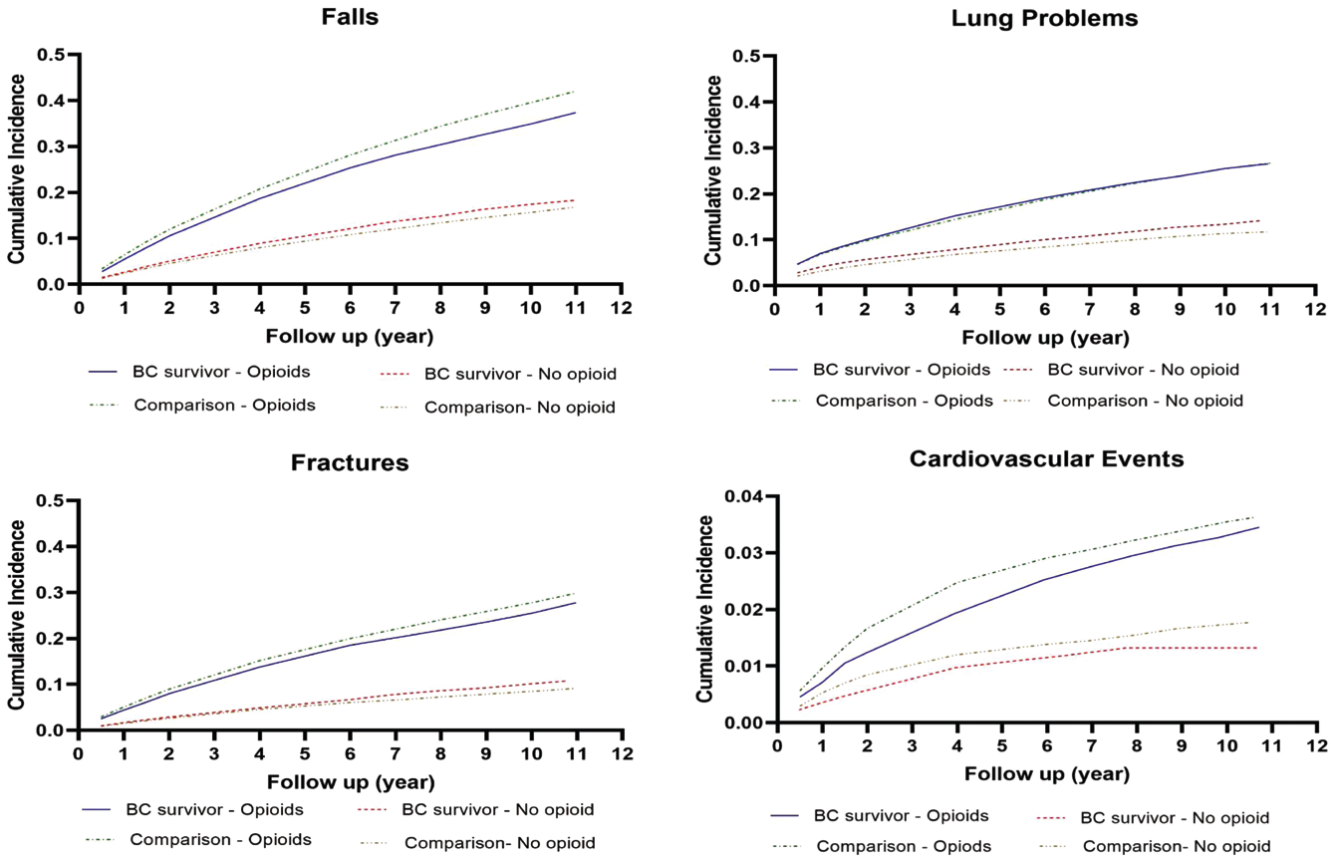
Cumulative incidence of adverse outcomes in breast cancer survivors and matched comparisons by opioid use during follow-up.

The multivariable Cox proportional hazard model determined the risk of falls was 12% higher and fractures was 56% greater in breast cancer survivors who used opioids versus comparison subjects unexposed to opioids (adjusted HR, 95% CI, 1.12 [1.07-1.17], and 1.56 [1.48-1.65]), respectively (Figure 3). The risk of all adverse outcomes was also statistically significantly higher in the comparison subjects who used opioids vs comparison unexposed to opioids (*P <* .01 for all outcomes). These risks were adjusted for baseline age; race/ethnicity; income; BMI; comorbidity status (ECI); depression; anxiety; use of psychiatric drugs; concurrent use of other pain medications (NSAIDs, cannabinoids, gabapentin); and history of these events prior to index date. In corresponding cumulative incidence graphs adjusted for the aforementioned covariates, the incidence of all adverse outcomes rose more steeply over the follow-up period in women exposed to opioids, regardless of breast cancer survivor or comparison cohort status (Figure 4).

### Sensitivity analysis—breast cancer survivor group

In sensitivity analyses restricted to the breast cancer survivors, those who were White non-Hispanic (52%) were more likely to use opioids than other groups (eg, 22% Hispanic; 14% Asian/Pacific Islander; 12% African American/Black patients; *P* < .001) (Table S2). Among breast cancer survivors, opioid use was higher in those who lived in neighborhoods with lower geocoded income; with more comorbidities; have obesity; and were former smokers (*P* < .001 for all variables, Table S2). Regarding tumor characteristics, opioid use was more common in those who had regional-stage tumors; underwent mastectomy; and had chemotherapy (*P* < .001 for all variables, Table S3).

**Figure 5.**
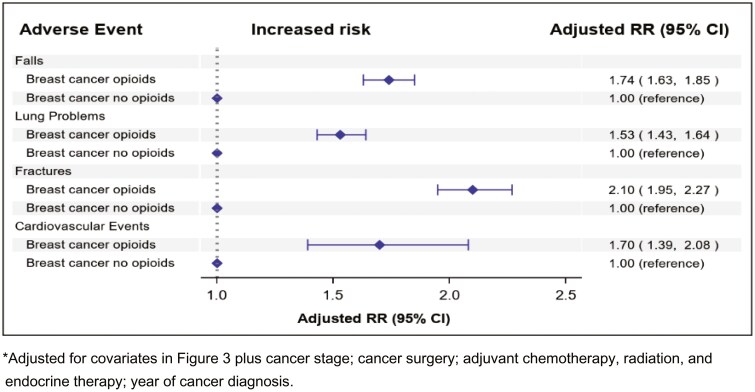
Adjusted risk of adverse outcomes by opioid use among breast cancer survivors.

In the multivariable analyses, opioid use beyond the first year of survivorship was associated with a 74% increased risk of falls (adjusted HR = 1.74, 95% CI, 1.63-1.85), while risk of fractures was nearly doubled (HR = 2.10, 95% CI, 1.95-2.27) compared with non-use (reference) (Figure 5). Opioid use was also associated with a 53% greater risk for lung problems (HR = 1.53, 95% CI, 1.43-1.64) and a 70% greater risk of cardiovascular events (HR = 1.70, 95% CI, 1.39-2.08). The multivariable model included the same covariates as in Figure 3 with additional adjustments for tumor characteristics, such as tumor stage, surgery type, adjuvant chemotherapy, radiation, and endocrine therapy [aromatase inhibitors and tamoxifen]; and year of cancer diagnosis.

## Discussion

This longitudinal cohort study of breast cancer survivors examined the adverse health risks related to opioid use in the years following active cancer treatment. To date, no studies have compared if opioid-related risks in breasst cancer survivors are similar to a matched group of women without cancer, to our knowledge. Opioid use was common in older women in general, regardless of whether they were breast cancer survivors or in the matched comparison group. However, important differences were seen in patterns of opioid use in the breast cancer survivor cohort versus their matched cancer-free comparisons. In this study, over 60% of breast survivors used opioids beyond their first year of survivorship forward, while 48% of the comparison subjects used opioids during the follow-up period. We observed that breast cancer survivors had greater opioid use and were prescribed stronger opioids, as assessed by daily maximum MME than women in the matched comparison cohort. Further, breast cancer survivors had a 12% greater risk of opioid-related falls and 56% greater risk of fractures than comparisons without opioid exposure.

Within the group of breast cancer survivors, about 11% had persistent opioids beyond the first year of survivorship. This is concerning because it is much higher than a SEER-based study that reported 2%-4% persistent opioid use among breast cancer survivors; this difference may partially be because our present study included a wider age range of adults, while the SEER-based study only included older Medicare beneficiaries aged over 65 years.^14,20^ Our finding of 11% with persistent use was also seen in the matched comparisons; this finding is consistent with a Centers for Disease Controls report that death rates attributed to prescription opioids, synthetic opioids, benzodiazepines, cocaine and heroin markedly increased by 260% in middle-aged women from 1997 to 2017.^5^

Opioid use was associated with a greater risk of falls, fractures, lung problems, and cardiovascular events in breast cancer survivors (Figure 5). Given that breast cancer survivors who are post-menopausal are often prescribed adjuvant aromatase inhibitors which may affect bone health, opioid use may further exacerbate the risk of fractures in this population.

The 2019 CDC prescribing guidelines for opioids state that their guidelines do not apply to patients undergoing active cancer treatment; rather, cancer pain management protocols should be based on American Society for Clinical Oncology^22,23^ and the National Comprehensive Cancer Network recommendations^24^ which advise restrained use of opioids to minimize potential dependence and addiction after active cancer treatment. Our results showed that 11% of breast cancer survivors had persistent use of opioids in the years following active treatment, which suggests opioid use occurs well beyond active cancer treatment to cope with residual cancer pain. Notwithstanding pain problems, this study is one of the few that quantified the other adverse health risks of opioid use, and suggests that optimal pain management with opioids requires thoughtful consideration of harms especially among cancer survivors, and furthermore, cancer survivorship care plans should specifically include monitoring of such use.

Strengths of this study include the longer follow-up period examined than in previous studies of opioid use; medication use ascertained from filled pharmacy prescriptions; the large diverse patient population; the wide range of ages; the comprehensive set of covariates; and use of the health plan’s NCI-SEER affiliated tumor registry that ensured accuracy of tumor characteristics and primary cancer treatment data. Our study is also the first to include an age-matched comparison cohort. Although opioid use was lower in the comparison group (48% vs 60% in breast cancer survivors), the overall high percentage of opioid use, as well as the elevated rates of adverse events in both groups, is striking and warrants further investigation and understanding of how opioid use and potential misuse or abuse is contributing to other adverse health events. Reasons why there was a high prevalence of opioid use in the matched comparison cohort is unclear. According to the Centers for Disease Control, fentanyl and other opioid use has increased by 53% from 2019 to 2020 in the general population aged 65 years and over.^25^

Certain limitations should be considered. Opioid use and dosage used were derived from pharmacy-filled prescriptions database, with an assumption that individuals took the medications as prescribed, which may not reflect the actual dose consumed. Thus, it is possible that patients may not be consuming the prescribed opioids as directed and potentially abuse them which would make the observed association even stronger. The findings in this insured population may not be generalizable to uninsured or underinsured populations, or to breast cancer survivors living in other countries without similar access to healthcare. Given that this study was based in an integrated healthcare setting with coordinated care, it is possible that patients cared for in other types of healthcare settings might have received more opioid prescriptions, and therefore the risks of adverse events associated with opioid use during survivorship period might be even stronger. Alternatively, among underinsured or uninsured patients, some patients might have not been able to get opioid prescriptions filled, thus, the associations might be attenuated in such populations. However, members of this healthcare system mirror the rest of southern California in terms of the distribution of race/ethnicity and socioeconomic status.^26^ Additionally, the association with risk of falls might also be partially attributed to menopause-related calcium loss that can exacerbate the risk of osteoporosis, which is further compounded by breast cancer treatments such as aromatase inhibitors; however, in the sensitivity analyses restricted to breast cancer survivors, the falls risk persisted even after adjusting for age and adjuvant endocrine therapy (Figure 5). We also could not perform any formal analysis on opioid use disorder as it was a rare event in this study; however, opioid use disorder might be underdiagnosed in general medical settings.

## Conclusion

In summary, opioid use was common in breast cancer survivors beyond the first year of cancer survivorship and was associated with increased risk for falls, lung problems, fractures, and cardiovascular events. For example, as breast cancer survivors who are post-menopausal are often prescribed adjuvant aromatase inhibitors which may affect bone health, opioid use may further exacerbate the risk of fractures in this population. Thus, clinicians need to weigh the risk and benefits of treating pain with opioids after active cancer treatment. Furthermore, the identification of the high rates of opioid use and adverse health events in both breast cancer survivors and the comparison cohort suggests a need for further study of both the physical and psycho-social needs of women in this demographic. Potential areas of exploration include the use of alternate pain management therapies such as acetaminophen and nonsteroidal anti-inflammatory drugs (NSAIDS); connecting patients with psychosocial support to educate patients about the risk of misuse and provide them with behavioral methods to manage pain control; other prescription medications such as antidepressants^27^; and implementing programs such as identifying opioid use disorder via electronic medical records in integrated healthcare systems to tightly monitor patients taking prescribed opioids to enhance safety of opioid use.^28^

## Supplementary material

Supplementary material is available at *The Oncologist* online.

oyae270__suppl_Supplementary_Tables_1-3

## Data Availability

The data underlying this article cannot be shared publicly due to the privacy of the Kaiser Permanente health plan members included in the study. A de-identified dataset can be shared upon reasonable request by writing to the corresponding author.

## Presentations

Abstract of preliminary results was presented at American Society of Clinical Oncology:

R Haque, LH Chen, J Shi, Z Gu, M Brady-Rogers, RT Chlebowski, R Hechter. Adverse health effects of opioids in survivors of breast cancer after active cancer treatment. J Clin Oncol Vol. 41, Suppl 16; Abstr e24145. 2023.
