# Supplementary material for: Opioid use and adverse health effects in breast cancer survivors
Source: Oncologist. 2024 Oct 14;30(3):oyae270. doi: 10.1093/oncolo/oyae270 (PMC11954500; doi:10.1093/oncolo/oyae270)
Supplement: oyae270__suppl_Supplementary_Tables_1-3 [file oyae270__suppl_supplementary_tables_1-3.docx]

Supplemental Table 1. ICD-9-CM and ICD-10-CM codes of study outcomes

|  | **ICD-9 Codes** | **ICD-10 Codes** |
| --- | --- | --- |
| **Cardiovascular events** |  |  |
| Cardiac arrhythmia | 427.9 | I49.9 |
| Stroke | 433.01, 433.11, 433.21, 433.31, 433.81, 433.91, 434.01, 434.11, 434.91 | I63.xx |
| Acute myocardial infarction | 410.90-410.92 | I29.xx |
|  |  |  |
| **Lung problems** |  |  |
| Chronic obstructive pulmonary disease [COPD] | 491.20, 493.20, 496 | J44.9 |
| Pneumonia | 486 | J18.9 |
|  |  |  |
| **Injuries** |  |  |
| Fractures | 733.1x, 733.81, 733.82, 800.xx-829.xx, 905.2-905.4, V54.10, V54.12-V54.16 | S12, S22, S32.0- S32.2, S32.3-S32.9, S52.x, S62, S72.x, S82.x |
| Falls | E880.0-E888.9 | W00-W19 |

Supplemental Table 2. Selected demographic characteristics at baseline by opioid use among breast cancer survivors diagnosed 2009-2019

|  | **Breast Cancer Survivors** | | | |  |
| --- | --- | --- | --- | --- | --- |
|  | **Total (N=33989)** | **Non-user (N=13678)** | **User (N=20311)** | ***P*-value*** | |
|  | N (%) | N (%) | N (%) |  | |
| **Age at index** |  |  |  | <0.001 | |
| <40 | 1142 (3.4) | 362 (2.6) | 780 (3.8) |  | |
| 40-64 | 17946 (52.8) | 7194 (52.6) | 10752 (52.9) |  | |
| 65+ | 14901 (43.8) | 6122 (44.8) | 8779 (43.2) |  | |
| **Race/ethnicity** |  |  |  | <0.001 | |
| Non-Hispanic White | 17030 (50.1) | 6458 (47.2) | 10572 (52.1) |  | |
| Non-Hispanic Black | 4176 (12.3) | 1402 (10.3) | 2774 (13.7) |  | |
| Hispanic | 7533 (22.2) | 2984 (21.8) | 4549 (22.4) |  | |
| Asian/Pacific Islander | 4802 (14.1) | 2620 (19.2) | 2182 (10.7) |  | |
| Mixed/other | 448 (1.3) | 214 (1.6) | 234 (1.2) |  | |
| **Geocoded median annual household income** | |  |  | <0.001 | |
| < $40,000 | 3979 (11.7) | 1375 (10.1) | 2604 (12.8) |  | |
| $40,001-$65,000 | 11399 (33.5) | 4175 (30.5) | 7224 (35.6) |  | |
| $65,001+ | 18588 (54.7) | 8113 (59.3) | 10475 (51.6) |  | |
| Missing | 23 (0.1) | 15 (0.1) | 8 (0.0) |  | |
| **Elixhauser Comorbidity Index** |  |  |  | <0.001 | |
| 0 | 2918 (8.6) | 1838 (13.4) | 1080 (5.3) |  | |
| 1 | 5583 (16.4) | 2719 (19.9) | 2864 (14.1) |  | |
| 2 | 6840 (20.1) | 2887 (21.1) | 3953 (19.5) |  | |
| 3 | 6155 (18.1) | 2396 (17.5) | 3759 (18.5) |  | |
| 4 | 4640 (13.7) | 1604 (11.7) | 3036 (14.9) |  | |
| *>*5 | 7853 (23.1) | 2234 (16.3) | 5619 (27.7) |  | |
| **Body mass index** |  |  |  | <0.001 | |
| <18.5 (Underweight) | 485 (1.4) | 260 (1.9) | 225 (1.1) |  | |
| 18.5 - 24.9 (Healthy) | 9751 (28.7) | 4514 (33.0) | 5237 (25.8) |  | |
| 25 - 29.9 (Overweight) | 10896 (32.1) | 4409 (32.2) | 6487 (31.9) |  | |
| 30 or more (Obese) | 12833 (37.8) | 4481 (32.8) | 8352 (41.1) |  | |
| Missing | 24 (0.1) | 14 (0.1) | 10 (0.0%) |  | |
| **Smoking history** |  |  |  | <0.001 | |
| Never | 23272 (68.5) | 9960 (72.8) | 13312 (65.5) |  | |
| Current | 1311 (3.9) | 485 (3.5) | 826 (4.1) |  | |
| Former | 7785 (22.9) | 2653 (19.4) | 5132 (25.3) |  | |
| Unknown | 1621 (4.8) | 580 (4.2) | 1041 (5.1) |  | |
| ^*^Chi-Square test (two-sided) |  | | | | |

Supplemental Table 3. Tumor characteristics and cancer treatments among breast cancer survivors diagnosed at 2009-2019 by opioid use status during study follow-up

|  | | **Opioid use during follow-up** | |  |
| --- | --- | --- | --- | --- |
|  | **Total (N=33989)** | **Non-user (N=13678)** | **User (N=20311)** | ***P*-value*** |
|  | N (%) | N (%) | N (%) |  |
| **SEER summary stage** |  |  |  | <0.001 |
| In situ | 7098 (20.9) | 3121 (22.8) | 3977 (19.6) |  |
| Localized | 18896 (55.6) | 7792 (57.0) | 11104 (54.7) |  |
| Regional | 7995 (23.5) | 2765 (20.2) | 5230 (25.8) |  |
| **Primary surgery** |  |  |  | <0.001 |
| Breast conserving surgery | 18709 (55.0) | 8346 (61.0) | 10363 (51.0) |  |
| Mastectomy | 14418 (42.4) | 4927 (36.0) | 9491 (46.7) |  |
| Primary treatment none/unknown | 862 (2.5) | 405 (3.0) | 457 (2.3) |  |
| **Adjuvant Hormonal Treatment** |  |  |  | <0.001 |
| No | 12826 (37.7) | 4923 (36.0) | 7903 (38.9) |  |
| Yes | 21163 (62.3) | 8755 (64.0) | 12408 (61.1) |  |
| **Adjuvant Chemotherapy** |  |  |  | <0.001 |
| No | 22444 (66.0) | 9760 (71.4) | 12684 (62.4) |  |
| Yes | 11545 (34.0) | 3918 (28.6) | 7627 (37.6) |  |
| **Adjuvant Radiation** |  |  |  | <0.001 |
| No | 18009 (53.0) | 6868 (50.2) | 11141 (54.9) |  |
| Yes | 15980 (47.0) | 6810 (49.8) | 9170 (45.1) |  |
| **Year of diagnosis**, n (%) |  |  |  | <0.001 |
| 2009-2010 | 5355 (15.8) | 1193 (8.7) | 4162 (20.5) |  |
| 2011-2012 | 5563 (16.4) | 1466 (10.7) | 4097 (20.2) |  |
| 2013-2014 | 5754 (16.9) | 1809 (13.2) | 3945 (19.4) |  |
| 2015-2016 | 6435 (18.9) | 2702 (19.8) | 3733 (18.4) |  |
| 2017-2018 | 7151 (21.0) | 3972 (29.0) | 3179 (15.7) |  |
| 2019 | 3731 (11.0) | 2536 (18.5) | 1195 (5.9) |  |
| *Chi-Square *P*-value (two-sided) | | | | |
